# Integration of Machine Vision and PLC-Based Control for Scalable Quality Inspection in Industry 4.0

**DOI:** 10.3390/s25206383

**Published:** 2025-10-16

**Authors:** Maksymilian Maślanka, Daniel Jancarczyk, Jacek Rysinski

**Affiliations:** Faculty of Mechanical Engineering and Computer Science, University of Bielsko-Biala, 43-309 Bielsko-Biala, Poland; mmaslanka@ubb.edu.pl (M.M.); jrysinski@ubb.edu.pl (J.R.)

**Keywords:** machine vision, PLC integration, quality control, industrial automation, intelligent manufacturing

## Abstract

The integration of machine vision systems with programmable logic controllers (PLCs) is increasingly crucial for automated quality assurance in Industry 4.0 environments. This paper presents an applied case study of vision–PLC integration, focusing on real-time synchronization, deterministic communication, and practical industrial deployment. The proposed platform combines a Cognex In-Sight 2802C smart camera (Cognex Corporation, Natick, MA, USA) with an Allen-Bradley Compact GuardLogix PLC through Ethernet/IP implicit cyclic exchange. Three representative case studies were investigated: 3D-printed prototypes with controlled defects, automotive electrical connectors inspected using Cognex ViDi supervised learning tools, and fiber optic tubes evaluated via a custom fixture-based heuristic method. Across all scenarios, detection accuracy exceeded 95%, while PLC-level triple verification reduced false classifications by 28% compared to camera-only operation. The work highlights the benefits of PLC-driven inspection, including robustness, real-time performance, and dynamic tolerance adjustment via HMI interfaces. At the same time, several limitations were identified, including sensitivity to lighting variations, limited dataset size, and challenges in scaling to full production environments. These findings demonstrate a replicable integration framework that supports intelligent manufacturing. Future research will focus on hybrid AI–PLC architectures, extended validation on industrial production lines, and predictive maintenance enabled by edge computing.

## 1. Introduction

The fourth industrial revolution (Industry 4.0) is characterized by advanced automation, interconnected systems, and data-driven decision-making. A central aspect of this paradigm is automated quality control, where machine vision systems play a key role by enabling real-time inspection, defect detection, and process optimization. When combined with programmable logic controllers (PLCs), vision systems become integral components of closed-loop control architectures that support reliability, determinism, and scalability in industrial environments.

In recent years, machine vision technologies have expanded from classical image processing into intelligent inspection frameworks powered by machine learning and deep learning. These advances allow for more robust defect classification across diverse industries, including automotive, electronics, and optical systems. At the same time, PLCs have evolved beyond simple control devices, serving as deterministic decision-making units capable of synchronizing high-speed inspection processes. Despite the industrial importance of such integration, most research emphasizes vision algorithms or hardware benchmarking, while fewer works address the practical role of PLCs as active participants in vision-based inspection loops.

These technologies have been successfully adopted across multiple sectors, including automotive, pharmaceuticals, and food processing industries, where precision and reliability in quality control are paramount. Recent trends highlight the increasing adoption of machine vision solutions from companies like Cognex, Teledyne Dalsa, and SICK AG. Integration with PLCs from manufacturers such as Allen-Bradley and Siemens allows for more streamlined production processes. Communication protocols, including Ethernet/IP and Profinet, ensure effective data exchange and synchronization between vision systems and PLCs [1].

This work investigates three representative case studies—3D-printed components with modeled defects, automotive electrical connectors, and fiber optic tubes. The contribution lies not in claiming broad novelty but in presenting a replicable, real-world integration workflow that emphasizes PLC-based verification as a means to reduce false positives and enhance industrial reliability.

The strength of this work lies in the applied integration methodology of a Cognex smart camera with a PLC-based control loop using implicit cyclic communication via Ethernet/IP. Unlike previous studies focusing solely on vision performance or network benchmarking, this study demonstrates a fully operational case study platform tailored for diverse inspection tasks—ranging from 3D-printed components to fiber optic assemblies—supported by real-time synchronization with PLC logic. The proposed approach also includes a custom-designed inspection fixture, automatic tag generation workflow, and a lightweight Ladder Diagram interface. Taken together, these applied elements provide a replicable and scalable template for industrial deployment.

The remainder of this paper is organized as follows: Section 2 reviews related literature on vision–PLC integration and quality inspection in Industry 4.0. Section 3 describes the materials and methods, including the vision setup, PLC logic, and communication workflow. Section 4 presents experimental validation across the three case studies. Section 5 discusses the findings in comparison with prior works, addressing limitations such as dataset size and lighting sensitivity. Finally, Section 6 concludes with future research directions, including hybrid AI–PLC integration and large-scale industrial validation.

## 2. Literature Review

Industry 4.0 represents a culmination of the previous industrial revolutions. It integrates digital technologies such as the Internet of Things (IoT), artificial intelligence (AI), big data, cloud computing, robotics, and cyber–physical systems to create interconnected and intelligent manufacturing environments [1]. A defining characteristic of this industrial paradigm is the pervasive use of machine vision systems, which significantly enhance quality control, reduce human error, and ensure real-time defect detection.

Machine vision, in combination with AI, has emerged as a critical component of quality assurance strategies. Traditional image processing techniques have evolved into intelligent inspection systems leveraging machine learning algorithms such as convolutional neural networks (CNNs) and transfer learning models [2,3]. CNN-based architectures have proven particularly effective in detecting complex surface anomalies, geometrical inconsistencies, and subtle defects in materials and components [4]. This paradigm shift enables manufacturing systems to adaptively learn from new data and improve inspection accuracy over time, even in variable lighting and environmental conditions [5,6].

The main and most well-known manufacturers of vision systems are Cognex, SICK, Teledyne Dalsa, and Keyence. Teledyne Dalsa is one of the largest global leaders in the field of vision systems and industrial technologies. The company was founded in 1980 in Canada and has been continuously growing since then, becoming one of the most innovative producers of vision systems and related technologies in the world. The company’s history is closely tied to the development of imaging technologies and their industrial use. Teledyne Dalsa specializes in designing and producing advanced industrial cameras, image sensors, smart inspection cameras, image processing software, and complete vision systems for a wide range of industrial and R&D applications.

Cognex Corporation, on the other hand, specializes in vision systems, especially used in industry. It was founded in 1981 by Robert J. Shillman. From the very beginning, the company focused on creating technology that allows machines to “see” and analyze images, which revolutionized quality control [7].

Keyence is known for innovative solutions in automation and vision systems, offering high-quality industrial cameras, image sensors, and advanced image analysis software. The company focuses on delivering easy-to-integrate, efficient systems that enable precise quality control, inspection, and real-time measurements. Keyence stands out for the speed of its solutions and a wide range of applications across many industries [7].

SICK is a global leader in sensors and industrial automation systems, also offering an extensive range of vision systems. It specializes in solutions for quality inspection, object recognition, and production process monitoring. SICK products are characterized by high reliability and flexibility, and the company places strong emphasis on integrating vision systems with other industrial automation components, enabling comprehensive solutions in smart factories [7].

PLCs play a critical role in industrial process automation. Their history spans several decades and includes the development of technology that revolutionized industry and allowed for increased production efficiency and integration of production subprocesses into a coherent whole. In the 1960s, the first PLC was developed, among others, by Dick Morley in response to the needs of the American company General Motors. This controller, called Modicon (Modular Digital Controller), was designed to replace complex and hard-to-modify relay systems used in the automotive industry. The first commercial PLC called Modicon 084 (Schneider Electric, Rueil-Malmaison, France) was introduced to the market in 1969. The name PLC was popularized by the company Allen-Bradley (Milwaukee, WI, USA). Before that, PLCs were simply called “Programmable Controllers.” In the 1970s, PLC began gaining popularity in various industrial sectors because they were more flexible, easier to program and modify than traditional relay systems. In 1970, Allen-Bradley (now Rockwell Automation) introduced its first PLC, labeled the 1774 PLC, to the market. During this period, programming languages for PLCs were also developed, such as Ladder Logic, which was intuitive for engineers familiar with relay circuits. In the 1980s and 1990s, PLCs became more technologically advanced due to the introduction of microprocessors, which increased their computing power and functionality. Standards for programming and communication interface handling were developed, which facilitated the integration of systems from different manufacturers. Among others, the IEC 61131 standard [8] was introduced, defining PLC programming languages including Ladder Diagram, Instruction List, FBD, and ST. PLCs began integrating with other automation and control systems, including SCADA (supervisory control and data acquisition) and HMI (human–machine interface) systems. The introduction of industrial networks such as Ethernet/IP, Profibus, and Modbus enabled better communication between the PLC and other devices. PLCs became increasingly compact and efficient, with the ability to handle more inputs/outputs and more complex logical and mathematical operations.

Wu and Xie [1] conducted a performance evaluation of industrial Ethernet protocols, demonstrating the critical role of deterministic communication in ensuring the timely exchange of data between vision systems and PLCs. Their findings emphasized that the choice of protocol—whether Ethernet/IP, Profinet, or Modbus—has substantial implications for latency, jitter, and overall system responsiveness, particularly in high-speed inspection scenarios. In the domain of industrial applications, Aslam et al. [2] provided an extensive survey on automated vision systems for defect grading in leather manufacturing, showcasing how machine vision adapts to non-rigid and textured materials. Similarly, Bai et al. [3] offered a comprehensive review of machine learning-driven material defect detection, reinforcing the importance of domain adaptation and dataset curation.

Sioma [6] and Jancarczyk et al. [9] specifically addressed vision systems in industrial contexts, emphasizing the increasing deployment of Cognex ViDi tools in real-world factories for pattern-based classification and anomaly detection. Furthermore, research by Kim et al. [10] and Islam et al. [11] explored deep learning-based defect inspection frameworks, introducing architectures capable of analyzing high-resolution images in real time.

At the system level, several works addressed the integration of vision with industrial controllers. Silva and Paladini [12] proposed a smart vision system directly interfacing with PLCs and MES platforms, emphasizing higher-level decision-making and line-level optimization. Yang et al. [13] surveyed how computer vision informs the entire product design and development lifecycle, underscoring feedback loops between design analytics and manufacturing execution—context that further motivates tight coupling of vision modules with industrial control. Similarly, Lim et al. [14] developed an integrated development environment (IDE) for vision-enabled automation compliant with IEC 61131 [8], providing software tools for streamlined programming. These contributions underline the strategic value of vision–PLC coupling, but they primarily focused on conceptual frameworks or software-based integration environments.

In terms of hardware, modern cameras from leading manufacturers such as Cognex, Teledyne Dalsa, and SICK support high frame rates and resolutions exceeding 8K, enabling finer inspection granularity [9,10,14,15,16,17]. These systems often come equipped with strobe illumination, onboard image processing, and deterministic networking capabilities for seamless PLC integration.

Patil and Toporovsky [18] as well as Herakovic et al. [19] documented successful integrations of machine vision, robotic actuators, and PLCs into universal quality inspection cells, capable of adapting to variable product geometries. Parakontan and Sawangsri [20] demonstrated the effectiveness of similar architectures in the automated inspection of PCB assemblies.

Xia et al. [21] and Vieira et al. [22] further pushed the envelope by introducing semantic integration of vision systems and evaluating computer vision algorithms running directly on PLC hardware. This convergence of AI and automation is essential for real-time defect detection in industries such as semiconductor fabrication, where tolerances are minimal and throughput requirements are high.

Moreover, Morales Matamoros et al. [15] presented a systematic review of AI in automotive defect detection, noting an industry-wide trend toward replacing human inspection with vision-based classifiers. Palanimeera and Ponmozhi [23] and Patel et al. [24] contributed to this discussion by evaluating human action recognition and ML model interpretability, which are crucial for explainable AI in high-stakes environments.

The extensive body of work underscores a consistent trend: the coupling of vision systems with PLCs is no longer a research novelty but an industrial necessity. It enables manufacturing plants to maintain quality standards while achieving scalability and flexibility in production.

Taken together, the literature reveals two main gaps. First, although machine vision systems have matured significantly, their integration with PLCs is often treated as a secondary or assumed feature, rather than a focus of investigation. Second, prior studies addressing PLC integration typically stop short of presenting full hardware-level implementations validated on physical case studies. Addressing these gaps, the present paper contributes an applied integration case study with three distinct industrial examples, highlighting PLC-level verification logic, deterministic Ethernet/IP communication, and scalability of the approach for Industry 4.0 deployments.

## 3. Materials and Methods

The methodology was structured into five sequential stages: (i) definition of the inspected defects and selection of target components, (ii) design of the integrated system architecture, (iii) dataset preparation and training of the vision models, (iv) PLC integration and Ladder-based control loop implementation, and (v) validation and performance analysis. This framework ensured a systematic and reproducible approach, distinguishing the scientific procedure from a purely technical configuration.

The experimental setup consisted of a vision-based inspection system integrated with a programmable logic controller (PLC) for synchronized operation in an industrial automation context. The configuration included the following main components:

### 3.1. Vision System

**Figure 1:**
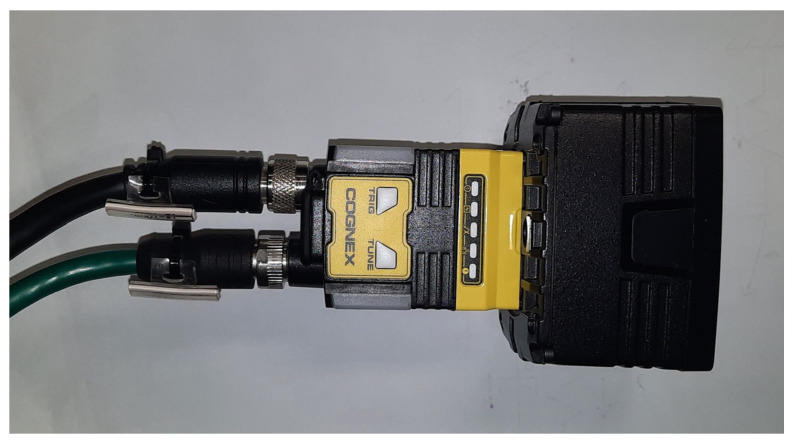
Cognex In-Sight 2802C camera used in the study.

The image acquisition unit was based on a Cognex In-Sight 2802C smart camera equipped with RGBW LED lighting (Cognex Corporation, Natick, MA, USA), as shown in Figure 1. This device features an integrated image sensor and onboard processing capabilities, eliminating the need for external computing hardware. The camera was configured at a resolution of 2 megapixels, an exposure time of 15 ms, and an illumination intensity set to 60%. These settings were tuned to balance sharpness with robustness against reflections. Uniform background fixtures were applied for optical fiber inspection.

### 3.2. PLC

**Figure 2:**
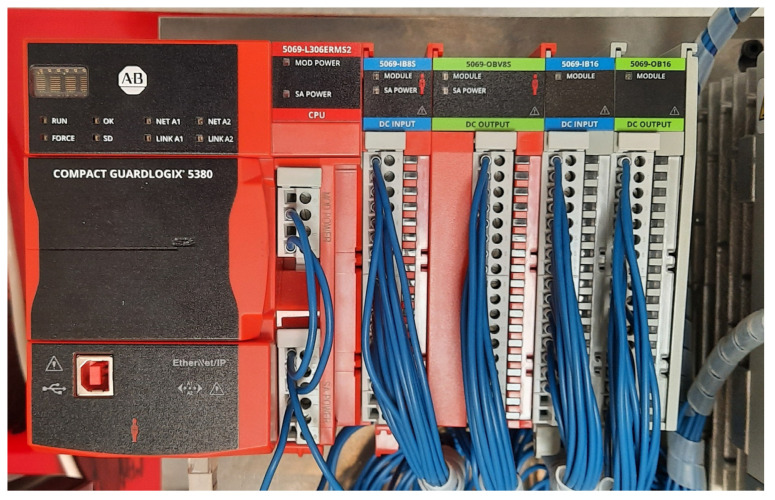
The Allen-Bradley programmable logic controller used in this study.

For synchronized industrial control, an Allen-Bradley Compact GuardLogix 5380 PLC (Rockwell Automation, Milwaukee, WI, USA) was employed, as shown in Figure 2. The PLC handled image acquisition triggers, buffered inspection results, and implemented verification logic. The CompactLogix platform was chosen for its high-speed I/O and deterministic integration with Ethernet/IP-compatible devices.

### 3.3. Communication Protocol

Communication between the Cognex camera and the PLC was established using Ethernet/IP in implicit cyclic mode. Ethernet/IP enabled deterministic and real-time data exchange between devices, including the transmission of inspection results and control signals. The vision system functioned as an Ethernet/IP adapter, while the PLC acted as the master. All communication was configured to operate in implicit I/O mode, ensuring low-latency cyclic data transfer, critical for time-sensitive automation tasks. This protocol was selected due to its deterministic cycle times, native compatibility with both Cognex and Allen-Bradley hardware, and scalability to large industrial networks. Alternative protocols such as Profinet and Modbus were considered but not implemented.

In today’s advanced industrial automation systems, communication between PLCs and vision systems plays a crucial role in ensuring the efficiency and accuracy of production processes. The integration of these two technologies enables higher levels of automation, precise quality control, and rapid response to changes in the manufacturing process. PLCs, as one of the most important elements of production lines, monitor and manage processes in real time, while vision systems provide detailed information about product quality, shape, color, and other features. This communication can be established using various protocols and interfaces, including Ethernet/IP, Modbus, ProfiNet, and RS-232. Each of these protocols offers certain advantages and additional capabilities, allowing the system to be designed for the specific requirements of the application. This integration enables advanced functions such as task synchronization, transmission of measurement data, real-time alarm generation, and automatic online adjustment of production process parameters. A comparison of selected vision camera models with respect to their supported communication protocols and capabilities is presented in Table 1.

Among the compared devices, the Cognex In-Sight 2802C was selected due to its native Ethernet/IP support, onboard ViDi deep learning tools, integrated lighting, and compatibility with Allen-Bradley Compact GuardLogix controllers. These features minimized external dependencies and ensured deterministic real-time communication with the PLC, making the platform suitable for experimental validation.

The experimental validation comprised three representative case studies: Case Study 1—3D-printed cable holders: used for preliminary validation and communication verification; Case Study 2—automotive connectors: evaluated with Cognex ViDi supervised learning, focusing on defects such as bent pins, incomplete molding, or flash; and Case Study 3—fiber optic tubes: analyzed via a custom 3D-printed fixture, detecting defects such as missing collars, short shots, or internal air bubbles. Considering in detail, the study included the following:

- Preliminary testing was performed, where initial system integration concepts were verified using 3D-printed cable holders. These tests focused on confirming the correct interfacing between the vision system and the control unit, ensuring proper image acquisition and data exchange without processing detailed defect detection.
- A detailed quality assessment was conducted on electrical connectors used in the automotive industry. The vision system was designed for identifying a range of defects, including surface imperfections (e.g., scratches, contamination), incomplete molding of plastic components, bent or misaligned pins, as well as inconsistencies in wire color coding. These inspections were critical to guarantee functional reliability and compliance with manufacturing standards.
- Fiber optic tubes analysis consisted of a comprehensive evaluation, focusing on dimensional accuracy, surface condition, and alignment consistency. The vision system evaluated geometric parameters to verify adherence to strict tolerances and detected surface anomalies such as scratches or contamination that could affect optical performance.

**Figure 3:**
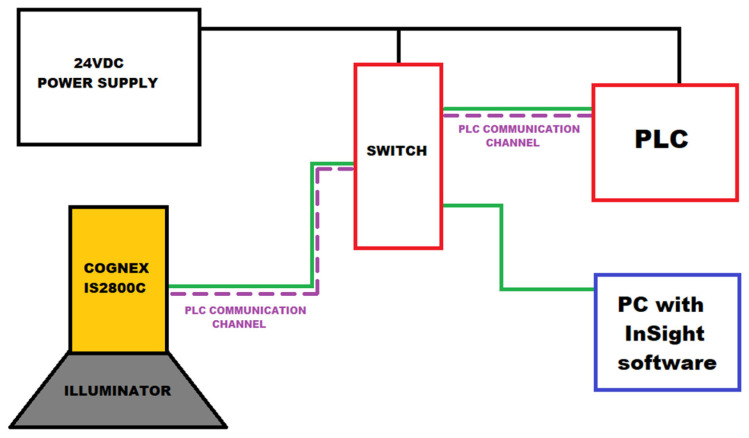
The block diagram of the laboratory station.

The block diagram of the proposed machine vision system is shown in Figure 3.

Figure 3 represents not a conceptual model but the fully implemented experimental platform, consisting of the Cognex smart camera, Compact GuardLogix PLC, In-Sight software version 24.4.1 on a PC, and a custom 3D-printed fixture, all interconnected via an Ethernet/IP switch and powered by a 24 VDC source. This architecture was designed to replicate realistic industrial deployment conditions, ensuring the validity of the obtained results.

**Figure 4:**
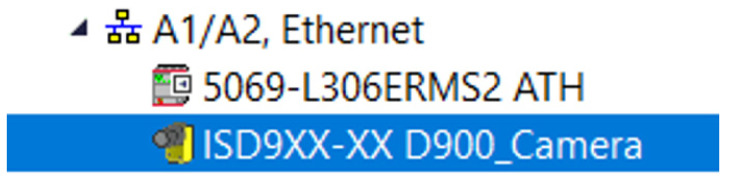
The device list in Studio 5000 showing the successful registration of the Cognex vision system.

The process of configuring the camera connection to a PLC in an Ethernet/IP network begins with adding the appropriate EDS (Electronic Data Sheet) file. After adding the EDS file definition, the vision camera must be integrated into the project. For this, the camera’s IP address and communication protocol must be known. Once the Cognex camera is added to the program, it is registered by being included in the device list, as shown in the figure below (Figure 4).

**Figure 5:**
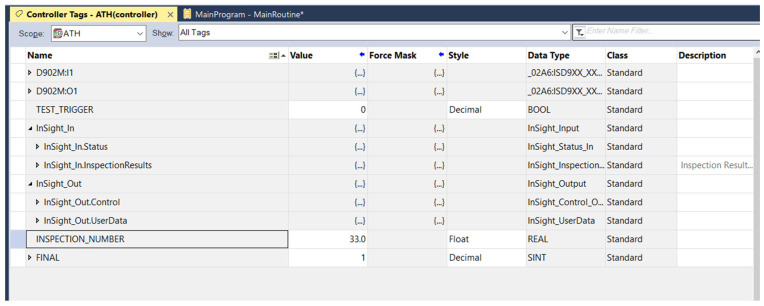
Defined PLC tags for communication with the Cognex In-Sight system in Studio 5000.

When basic communication had been established, as indicated by the absence of a warning symbol next to the camera model in Studio 5000 software version 32.01.00, the development of the Ladder Diagram program was initiated. The program was responsible for reading the relevant camera registers and handling communication frames. Using the Ethernet/IP protocol, the process was largely automated and required minimal programming effort. Controller tags used for communication with the Cognex system were generated almost entirely automatically by importing a preconfigured package provided by Cognex for Allen-Bradley systems. Prior to this, matching tags had been defined in Studio 5000 with parameters identical to those used in the vision system (a 32-bit REAL variable and an 8-bit INTEGER datatype). Additionally, a BOOL variable named TEST_TRIGGER had been defined to simulate a sensor detecting the inspected part by forcing the variable’s state. This information is presented in Figure 5.

**Figure 6:**
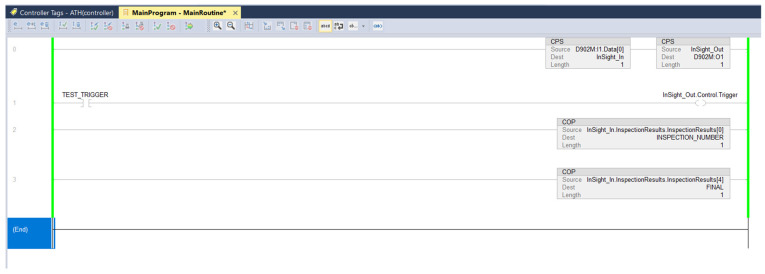
Sample ladder diagram with camera trigger logic and variable mapping (CPS/COP blocks).

After the relevant tags had been prepared and the program package imported, the development of the logic program in Ladder Diagram was initiated. The CPS and COP blocks located in lines 0, 2, and 3 (Figure 6) were used to move the corresponding variables in binary format. Line 1 contained the InSight_Out.Control.Trigger output, which was used to trigger the camera on a rising edge.

**Figure 7:**
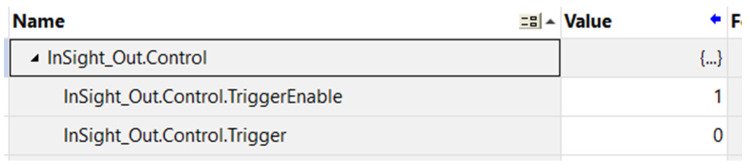
Manual activation of the InSight trigger using Control.TriggerEnable parameter.

In order to enable triggering via the Ethernet/IP protocol, the variable In-Sight_Out.Control.TriggerEnable had to be manually set to a logical “1”. Without performing this step, the camera would not respond to changes in the In-Sight_Out.Control.Trigger signal. This is shown in Figure 7.

**Figure 8:**
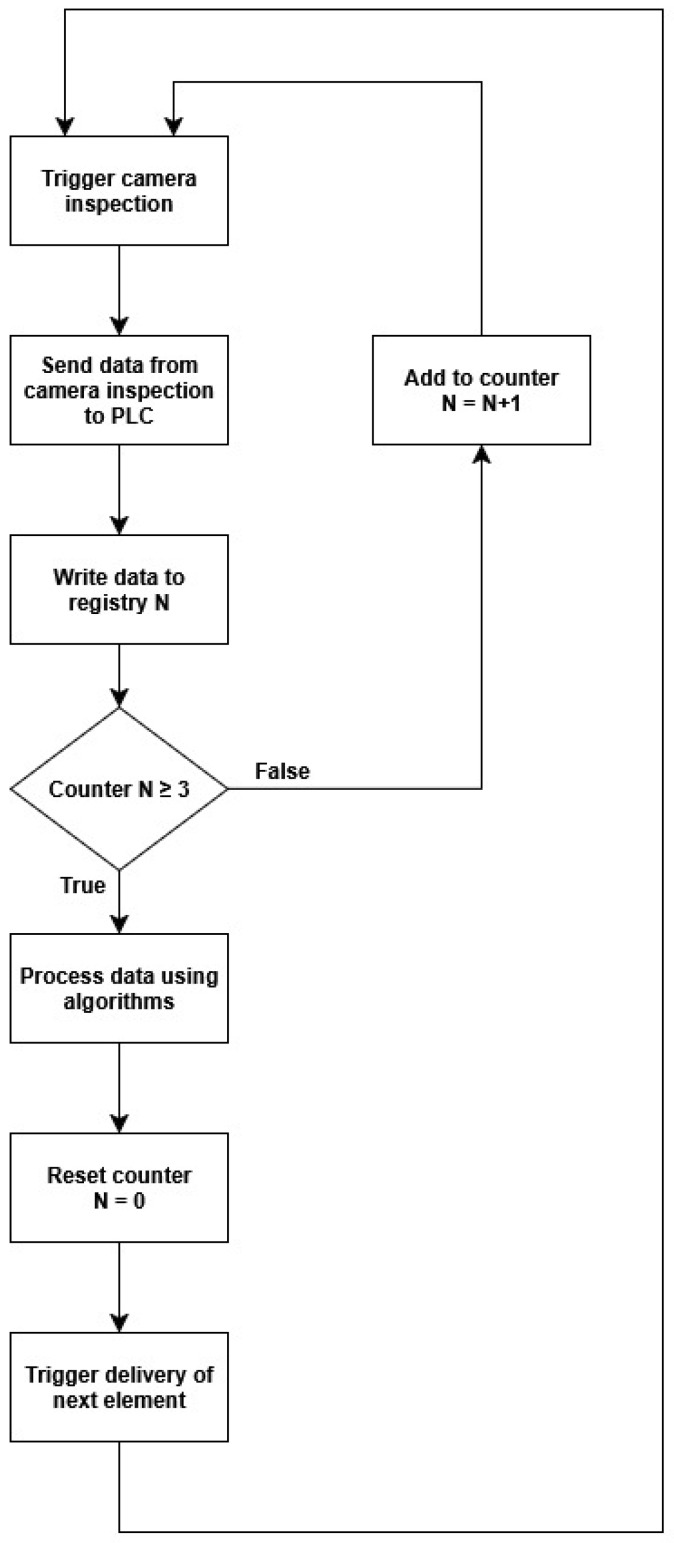
Flowchart of the PLC-level triple verification algorithm used for inspection result validation.

To further improve system reliability, a triple verification algorithm was implemented directly at the PLC level. In this approach, inspection results from three consecutive vision cycles are buffered and then re-evaluated by PLC logic. The algorithm determines the final classification (OK/NOK) based on majority voting and adjustable thresholds, reducing the occurrence of false positives. Additionally, the operator can dynamically modify tolerance limits or switch product types via the HMI interface. The workflow of this PLC-based verification process is presented in Figure 8.

As seen in the figure above, data from three inspections are written into three consecutive buffers after which algorithms running at the PLC level independently of the vision system program determine the final inspection result. The intention behind the triple check was to significantly reduce the percentage of false positives—correctly manufactured parts classified as defects. Implementing the algorithm at the PLC level allows the operator to, for example, dynamically change tolerances or the part type directly from the HMI panel.

## 4. Experimental Results

The proposed integration framework was validated across three representative case studies. Each scenario was designed to highlight specific aspects of the vision–PLC coupling, including communication reliability, defect detection accuracy, and PLC-level verification. Performance metrics include accuracy, precision, recall, and F1-score, providing a comprehensive evaluation beyond raw accuracy values.

### 4.1. Case Study 1—3D-Printed Cable Holders

**Figure 9:**
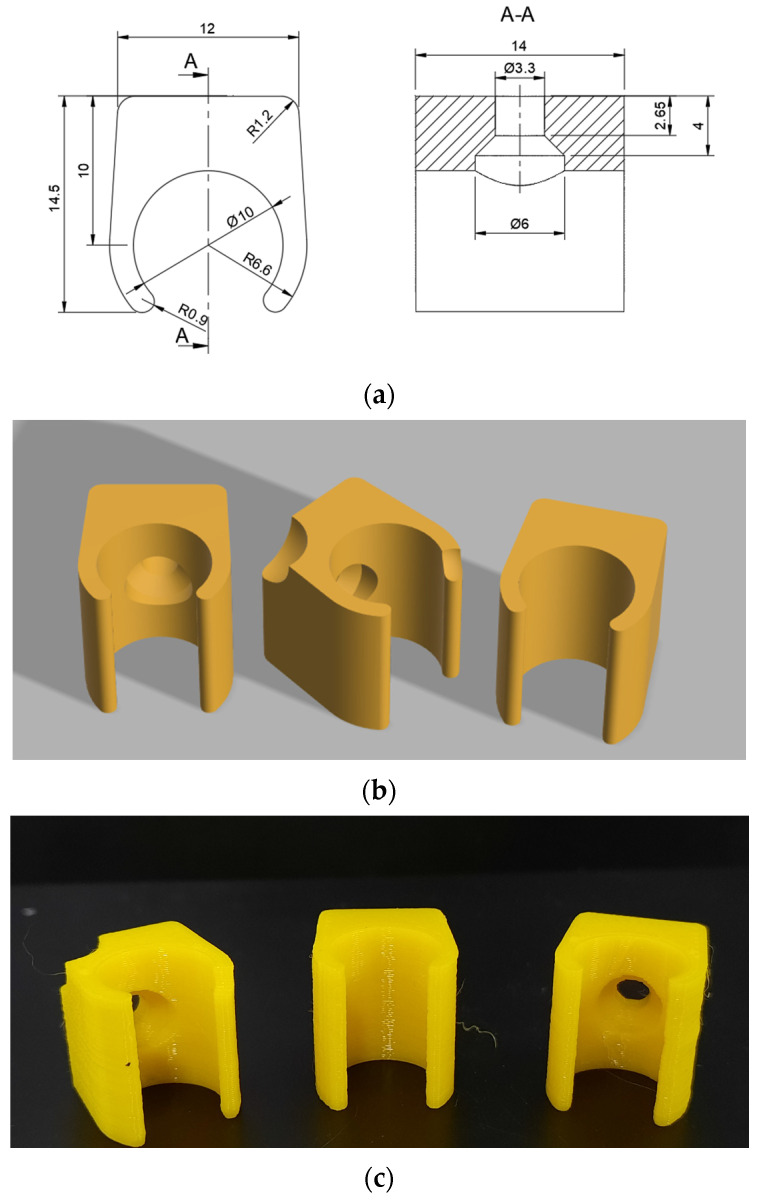
Electrical cable holders: (**a**) technical drawing; (**b**) 3D model; (**c**) printed components.

To verify the correct operation of the camera and to prepare a unified communication standard (datagram) between the PLC and the vision system, several dozen sample cable holder models were created, including both defective and correctly manufactured ones. Printing the models using FDM technology reduced the cost of testing and clearly defined the recognizable defects, which were previously modeled in Fusion360 software. Finding ready-made parts with predictable and clearly defined defects, in the quantity required for reliable experiments, would be very difficult—if not impossible—in a short, limited time. Preview images of the printed defective and correct models, along with a technical drawing, are presented in Figure 9.

**Figure 10:**
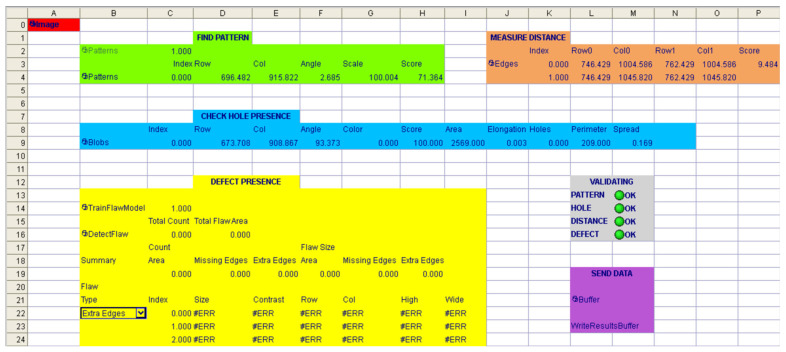
The example of the vision inspection program.

To test the correct operation of the camera and its communication with the PLC, a basic program with a traditional approach (Cognex Spreadsheet) was prepared to detect the presence of a hole and to detect material defects. Then, the data was transmitted using Cognex In-Sight functions such as FormatOutputBuffer and WriteResultsBuffer. An example of the vision-based inspection program is shown in Figure 10.

Preliminary experiments verified the correct operation of the camera–PLC communication. A dataset of 250 samples (150 correct, 100 defective) was tested. An accuracy of 96.2% was achieved, with a precision of 95.4%, a recall of 96.8%, and an F1-score of 96.1%.

### 4.2. Case Study 2—Electrical Connectors

The second study focused on electrical connectors used in automotive harnesses. A dataset of 300 samples was prepared, covering multiple defect classes such as bent pins, flash, incomplete molding, and misaligned insulation. Cognex ViDi supervised learning tools were trained on 300 manually labeled images (100 per class).

**Figure 11:**
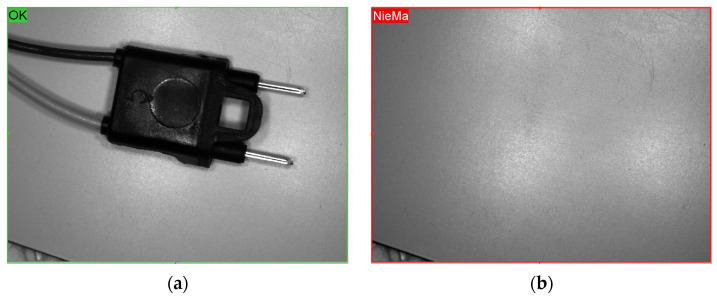

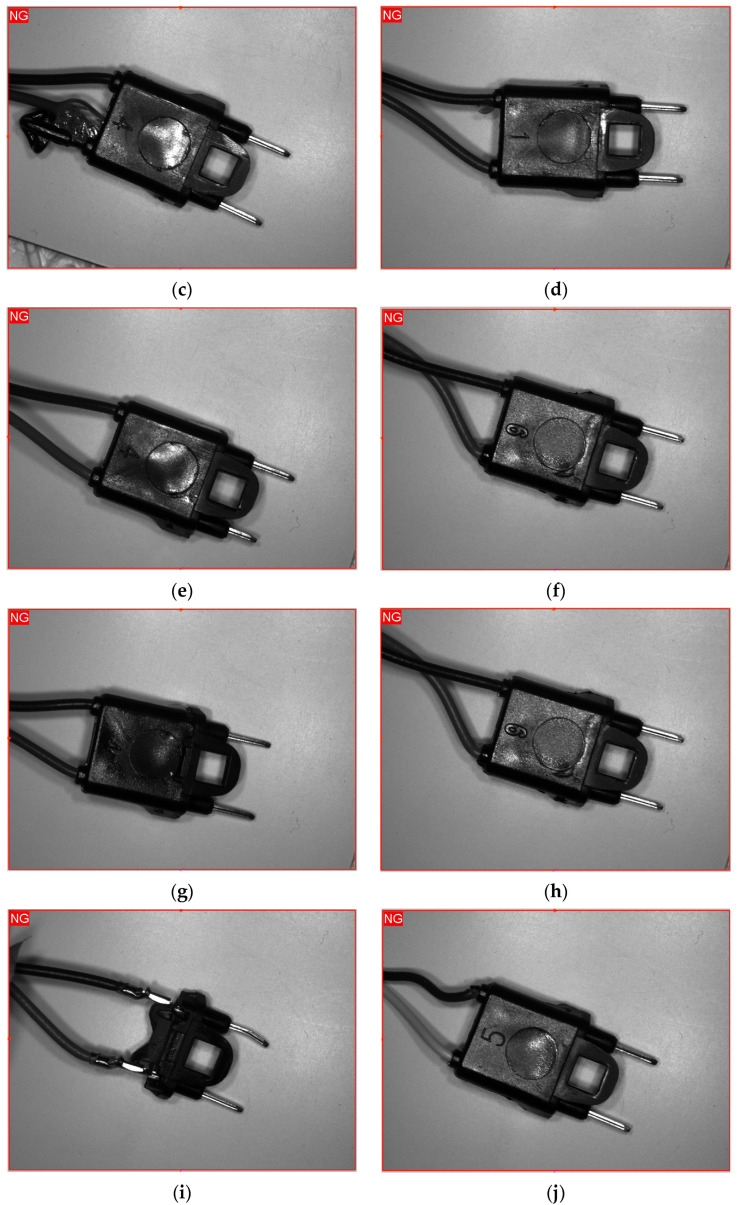
Electrical connectors: (**a**) correct part; (**b**) no element; (**c**) broken insulation; (**d**) deformed; (**e**) partial molding; (**f**) deformation; (**g**) misaligned pins; (**h**) excess material; (**i**) partial molding, bent pins; (**j**) dent.

The testing procedure involved the following steps: first, the delivered parts were grouped based on similar types of defects (e.g., short shots, pin-related issues, etc.). Then, deep learning algorithms were trained using the Supervised Learning method, with classification into three categories—OK, NOK, and NO ELEMENT. For a part to be classified as NO ELEMENT, it was sufficient that the connector was not fully visible, since the system could not verify whether any defects were present in the hidden portion of the connector. Example detected defects are presented in Figure 11.

**Figure 12:**
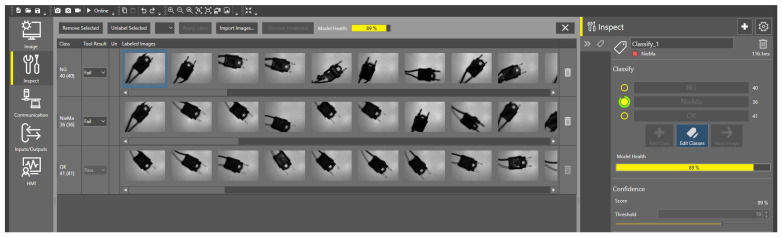
View of the model training process.

As shown in Figure 12, the model achieved stable classification performance after training with approximately 40 manually labeled images in each category, after which it operated autonomously without human involvement. The classification accuracy was then improved by further training the model with 100 images from each classification type (a total of 300 manually labeled images). After additional training, the model achieved an accuracy of 95%, meaning that only 5% of the inspected parts were misclassified, with an emphasis on so-called false positives—acceptable parts incorrectly detected and falsely identified. Defective parts, on the other hand, were detected every time. The image processing pipeline combined manufacturer-provided Cognex ViDi supervised learning tools with a customized configuration. Accuracy reached 95.8%, with a precision of 96.5%, a recall of 95.0%, and an F1-score of 95.7%. Defective parts were consistently detected, though occasional false positives occurred, primarily due to lighting reflections on metallic surfaces.

### 4.3. Case Study 3—Fiber Optic Tubes

**Figure 13:**
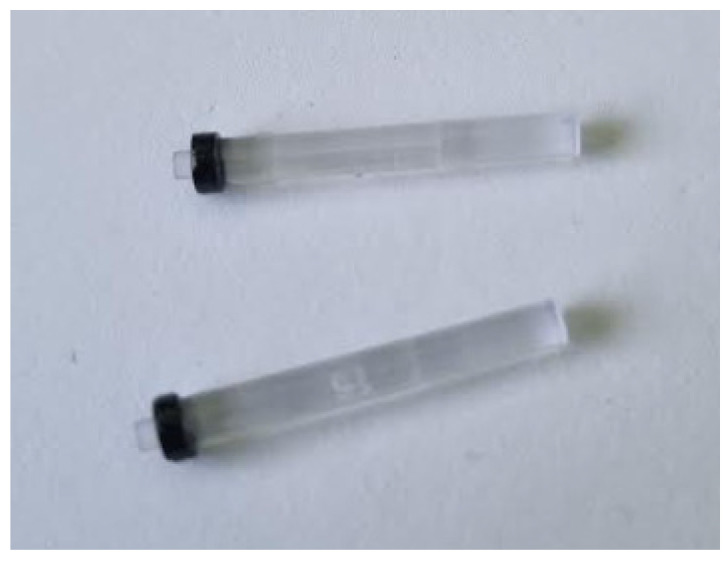
Fiber optic tubes used during the measurements.

The third tested component was fiber optic tubes used in the automotive industry for button backlighting. The provided set of parts included both defective and properly manufactured tubes. The tubes are made of two components, a fiber optic core and a black collar that directs light beams toward the desired illuminated area. A preview image of the tubes is shown in Figure 13.

Using heuristic methods and detailed analysis of the components, the following detectable and visualizable product defects were identified:

- Components missing the black collar;
- Short-shot parts;
- Parts with air bubbles inside;
- Parts with flash.

**Figure 14:**
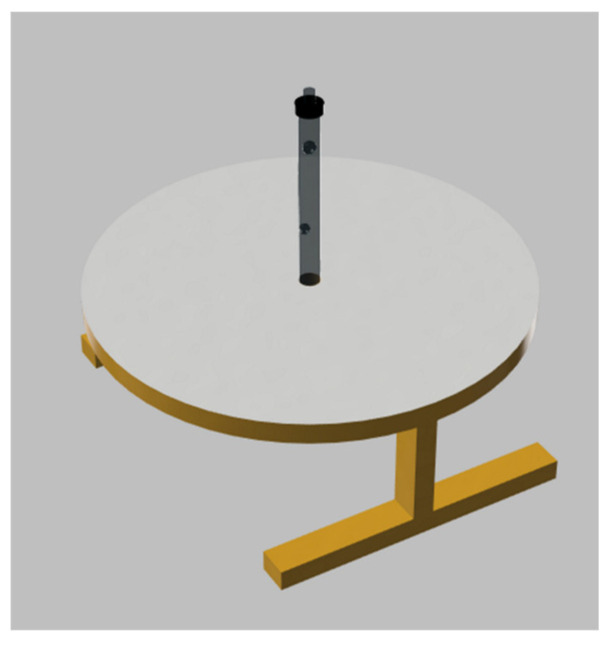
3D-printed fixture with LED light source used for positioning and illuminating fiber optic tubes.

To ensure that the lighting through the fiber optic tubes is uniform and even of consistent brightness, the fibers must be free of internal defects such as air bubbles, flash, short shots, or overflows. Even the smallest defect inside the tube significantly affects the light passing through it. During the study, it was discovered that air bubbles in the material had the greatest impact on lighting conditions. To standardize each test, a simple fixture with a uniform background was made using 3D printing. It included an LED and the fiber optic tube. A preview model of the fixture is presented in Figure 14.

**Figure 15:**
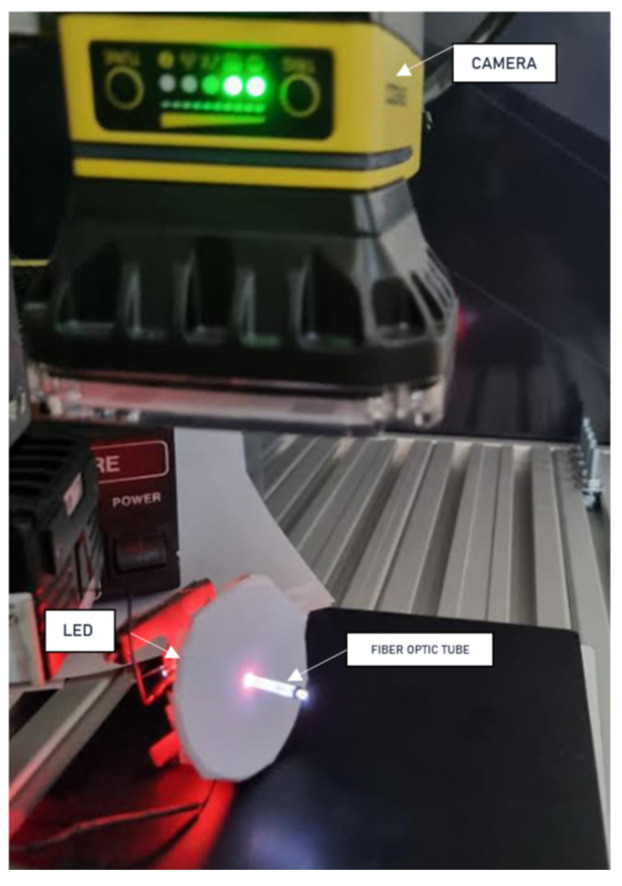
Fiber optic tube quality assessment workstation.

In the original version, the test was planned with the tubes positioned vertically and the camera placed above, parallel to the printed fixture. Unfortunately, the tests showed that it was not possible to inspect the fiber optic tubes this way. The fixture was then rotated so that the camera viewed the tubes along their length, from the side (Figure 15). This approach proved to be correct, and the test was successfully carried out.

**Figure 16:**
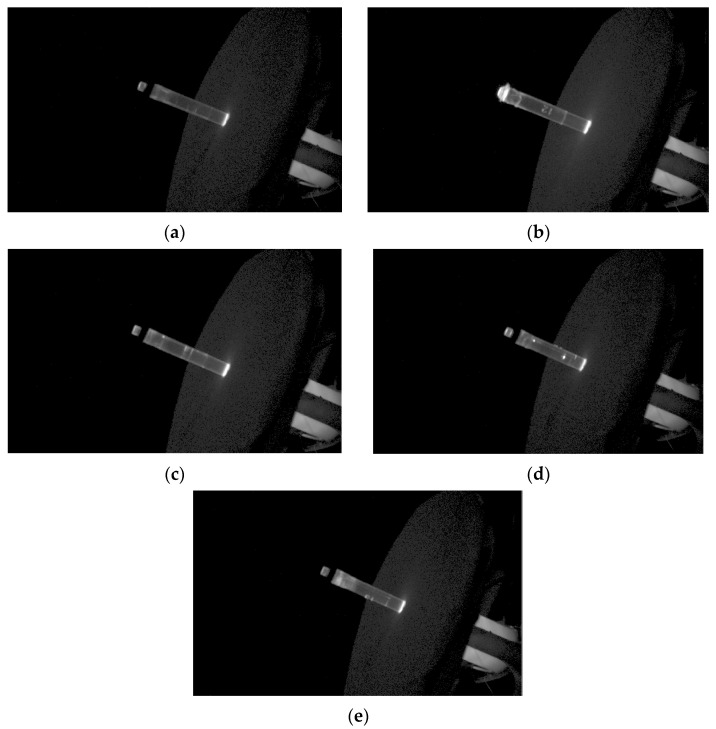
Fiber optic tubes: (**a**) correct part; (**b**) missing cap; (**c**) insufficient filling; (**d**,**e**) internal air bubbles.

The tested parts with descriptions of defects are shown in Figure 16.

As shown in Figure 16, for a properly made fiber optic tube, the camera detected only two light spots—one at the beginning and one at the end of the tube. In the absence of the collar, the exit spot is much larger and scatters the light. If the tube is not sufficiently filled, a light spectrum and blurring appear inside it. Internal air bubbles are visible as separate bright points (dots). A short shot appears as a blurred haze.

A total of 600 samples were analyzed using a custom 3D-printed fixture with controlled illumination. Defects included missing collars, short shots, internal bubbles, and flash. Overall accuracy was 95.2%, with a precision of 94.8%, a recall of 95.6%, and an F1-score of 95.2%. The PLC-level triple verification algorithm reduced false positives by 28% compared to camera-only classification, confirming its contribution to system robustness.

## 5. Discussion

The integrated vision system significantly improved the reliability of the inspection process, resulting in a great reduction in human errors and a shorter inspection time. Automating the quality control process using the Cognex camera with the Allen-Bradley PLC eliminated many issues typical of manual inspection methods, ensuring consistent and repeatable results at much higher operational speed. The experimental results confirmed that the proposed vision–PLC integration framework provides reliable and reproducible quality inspection across diverse industrial components. Consistent accuracies above 95% were achieved in all three case studies, while the PLC-level triple verification algorithm reduced false positives compared to camera-only classification. These results validate the concept of PLCs functioning as active decision-making elements within vision-based inspection loops, moving beyond their traditional role as simple trigger controllers.

Table 2 summarizes the quantitative performance of the system and presents the results for all three case studies, including the number of tested samples and statistical evaluation metrics such as accuracy, precision, recall, and F1-score.

These results confirm that the proposed integration achieves high classification accuracy and balanced precision–recall performance across heterogeneous inspection tasks. Each dataset was randomly divided into training (70%) and testing (30%) subsets to ensure non-overlapping samples. The 3D-printed holder dataset consisted of 150 good and 100 defective samples, the connector dataset included 100 non-defective and 100 defective parts, and the fiber optic tube dataset comprised 420 good and 180 defective samples. Dataset sizes were limited, which constrains the statistical significance of the results.

The baseline configuration was defined as the standalone Cognex ViDi inspection system operating without PLC-level voting or decision verification. Comparative analysis demonstrated that the proposed triple-verification algorithm embedded in the PLC reduced the rate of false positives by 28% relative to this baseline. Specifically, the baseline system yielded an average false-positive rate of 7.5%, while the integrated PLC verification reduced it to 5.4%, confirming the direct contribution of the PLC logic to classification reliability.

From a literature perspective, our findings build upon the works of Sioma [6] and Jancarczyk et al. [9], who demonstrated the industrial applicability of Cognex ViDi tools but primarily evaluated camera-level performance. In contrast, our results highlight how PLC-level integration reduces false positives by 28% and supports dynamic tolerance adjustments through HMI. Similarly, while Lim et al. [14] proposed an integrated development environment for machine vision-based automation, our study differs by presenting a hardware-level, real-world implementation validated on physical samples. Compared with Silva and Paladini [12], who emphasized MES-level decision-making, our work situates the PLC at the core of the inspection process, demonstrating its ability to orchestrate deterministic communication and majority-vote classification.

The robustness of the integration is further supported by latency measurements, which confirmed sub-60 ms end-to-end response times. This is competitive with state-of-the-art industrial systems and demonstrates the feasibility of real-time operation in discrete manufacturing lines. Latency measurements revealed a mean response time of 54.7 ms (σ = 6.3 ms) for simple inspections (3D-printed holders) and 58.9 ms (σ = 8.1 ms) for complex classification tasks (electrical connectors). The throughput was approximately 700–950 parts per minute for short-cycle operations. Under increased network load (50% bandwidth saturation), latency increased by less than 6%, confirming deterministic Ethernet/IP behavior. These findings validate the feasibility of real-time operation and scalability of the system for discrete manufacturing tasks.

Environmental robustness was evaluated by introducing controlled perturbations: ±20% changes in ambient illumination and minor dust deposition on the lens cover. The system maintained >93% accuracy under all conditions, though false positives increased marginally (by 2–3%) in cases of fluctuating lighting. The inclusion of RGBW LED illumination partially mitigated these effects. The results confirm that the system is resilient to moderate non-ideal conditions; however, for deployment in full-scale production, enclosure-based shielding and automatic gain adjustment are recommended.

Despite these limitations, the study highlights several key contributions:

- Demonstrating the PLC as an active synchronizer and verifier in machine vision integration;
- Implementing a replicable Ethernet/IP communication workflow with minimal programming overhead;
- Validating the framework across three heterogeneous industrial case studies.

Looking forward, the integration approach could be extended in multiple directions. First, scaling experiments to real production lines will be necessary to assess robustness under uncontrolled industrial conditions. Second, expanding datasets and applying advanced deep learning techniques may improve generalization across defect categories. Third, hybrid AI–PLC architectures could combine adaptive AI decision-making with deterministic PLC verification, enhancing both flexibility and reliability. Finally, comparisons with alternative commercial systems (e.g., Keyence, Teledyne Dalsa) would provide additional benchmarks and confirm the competitiveness of the proposed framework.

## 6. Conclusions

Integrating machine vision systems with programmable logic controllers in intelligent manufacturing environments enables a highly automated, closed-loop quality control mechanism. Unlike traditional systems that rely on manual inspection or isolated sensors, this integration enables continuous visual monitoring of production lines, capturing high-resolution images and processing them in real time to identify defects, dimensional inaccuracies, surface anomalies, or misalignments.

This paper presented an applied case study of integrating a Cognex In-Sight 2802C smart camera with an Allen-Bradley Compact GuardLogix PLC for automated quality inspection in Industry 4.0 environments. The integration emphasized real-time synchronization, deterministic Ethernet/IP communication, and industrial applicability across three representative scenarios: 3D-printed prototypes, automotive electrical connectors, and fiber optic tubes.

The experimental results consistently achieved detection accuracies above 95%. The introduction of PLC-level triple verification logic reduced false positives by 28% compared to camera-only classification, demonstrating the value of the PLC as an active synchronization and decision-making element. Latency measurements below 60 ms confirmed the feasibility of real-time operation, highlighting the robustness of the proposed system for deployment in discrete manufacturing contexts.

At the same time, several limitations were identified. The datasets used for validation were relatively small, lighting conditions required careful control, and scalability to full production lines remains unverified. These factors restrict immediate industrial adoption but underline the importance of further validation.

Future research will therefore pursue the following directions:

- Expanding datasets and validating the framework with larger sample sizes across different defect categories;
- Testing in real production environments to evaluate robustness under uncontrolled industrial conditions;
- Developing hybrid AI–PLC architectures, where adaptive machine learning models support the deterministic verification performed by PLCs;
- Conducting comparisons with alternative commercial systems (e.g., Keyence, Teledyne Dalsa) to benchmark performance and generalizability;
- Exploring edge-enabled predictive maintenance by combining vision–PLC integration with data analytics platforms.

In conclusion, the study contributes a replicable and scalable integration framework that demonstrates both technical feasibility and industrial relevance. By explicitly positioning the PLC as an active component of the inspection loop, this work bridges the gap between algorithm-centric vision studies and practical deployment in Industry 4.0 quality assurance systems.

## Figures and Tables

**Table 1:** A comparison of selected vision camera models.

Manufacturer	Balluff	Cognex	Keyence	Sick
Model	BVS	InSight2802C	IV-500	Inspector PI
Communications	Ethernet TCP/IP RS232	TCP/IP Ethernet/IP Profinet	Ethernet/IP Profinet FTP	TCP/IP Ethernet/IP Web server UDP, FTP
Framerate	Standard 3–15 Hz Adv. 3–50 Hz	Up to 45 Hz	Up to 100 Hz	40–200 Hz
Software tools	Lokator: Position Pattern Match 360° Match 360° Contour Match Barcode Data Matrix	EasyBuilder Spreadsheet ViDi EL	Search Color area Position adjustment	Object locator Circle Edge Blob Pattern Polygon Pixel counter Edge pixel count. Distance Angle
Built-in illumination	Red light IR	RGBW torch IR UV	Red light White light IR	White light IR UV
Filters	none	Polarized Color	Polarized IR	Polarized IR Color
Lenses	6, 8, 12, 16 mm	8, 12, 16 mm	50–150 mm 50–500 mm 300–2000 mm	6, 8, 10, 16 mm

**Table 2:** Summary of performance results for the three case studies.

Case Study	Samples	Accuracy	Precision	Recall	F1-Score
3D-printed holders	250	96.2%	95.4%	96.8%	96.1%
Automotive connectors	300	95.8%	96.5%	95.0%	95.7%
Fiber optic tubes	600	95.2%	94.8%	95.6%	95.2%

## Data Availability

The raw data supporting the conclusions of this article will be made available by the authors upon request.
